# Pregnancy Outcomes in Men and Women Treated With Teriflunomide. A Population-Based Nationwide Danish Register Study

**DOI:** 10.3389/fimmu.2018.02706

**Published:** 2018-11-23

**Authors:** Johanna Balslev Andersen, Julie Yoon Moberg, Tim Spelman, Melinda Magyari

**Affiliations:** ^1^Danish Multiple Sclerosis Registry, Department of Neurology, Copenhagen University Hospital, Rigshospitalet, Denmark; ^2^Department of Neurology, Danish Multiple Sclerosis Center, Copenhagen University Hospital, Rigshospitalet, Denmark

**Keywords:** teriflunomide, pregnancy-related outcomes, nationwide Danish register study, teriflunomide treated men and women, pregnancy-related outcomes for mother and child

## Abstract

**Background:** The majority of persons diagnosed with multiple sclerosis (MS) experience their first MS symptoms in the reproductive age. Teriflunomide (TFL, Aubagio), was first released in Denmark for relapsing-remitting MS in December 2013. TFL treatment is contraindicated in women of childbearing potential who are not using reliable contraception. TFL can be transmitted via semen and a low risk of male-mediated embryo-fetal toxicity is described.

**Objective:** To report pregnancy outcomes of TFL-treated women and partners to TFL-treated men: gestation week.

**Methods:** Prospective cohort study comparing pregnancy outcomes of TFL-treated men and women, matched on age at conception, 1:4 with controls from the general population. Data on TFL-treated patients treated 1st of January 2014–31st of December 2016 for at least 30 consecutive days prior to conception, and with conception occurring latest 2 years after treatment discontinuation were extracted from The Danish Multiple Sclerosis Registry and merged with several national reproductive registries. Logistic regression was used to analyse the association between TFL exposure and any adverse event.

**Results:** A total of 31 pregnancies were recorded, 13 women and 18 of partners to a TFL-treated man. All 18 partners of TFL-treated men completed their pregnancies: livebirth (18), gestation time >37 weeks (17), gestation time 33–36 weeks (1), normal birth weight (18), spontaneous and elective abortion (0), congenital malformation (plagiocephali) (1), normal delivery (14), induced delivery (2), cesarean section (2), Apgar score ≥7 (18). Among the 13 pregnancies in women exposed to TFL: elective abortion (11), spontaneous abortion (0), livebirth (2), gestation time >37 weeks (2), normal birth weight (2), congenital malformations (0), normal delivery (1), induced delivery (1), Apgar score ≥7 (2). The TFL group was associated with a 22% reduction in the odds of any adverse event relative to controls, although this association was not significant (OR 0.78; 95% CI 0.16–3.72, *p* = 0.753).

**Conclusion:** Pregnancy outcomes were consistent with those of the general population. The malformation reported of the partner to a TFL-treated man is comparable to the rate of plagiocephaly reported in Denmark.

## Introduction

Multiple sclerosis (MS) is a progressive chronic neurological disease manifesting in two main phenotypes, relapse-remitting (RR) and primary progressive course. Approximately 85% of MS patients have a RR onset with recurring acute relapses over a period of decades, transitioning at some point to a progressive, secondary phase with or without relapses (1). MS occurs more frequently in women than men, and the mean age in recent years at onset according to the Danish Multiple Sclerosis Registry (DMSR) is 35.5 years [standard deviation (SD) 10.5] for men and 34.6 (SD 10.7) for women, which means the majority of the patients is in their reproductive years at disease onset.

Teriflunomide (TFL) is a once-daily oral immunomodulator, which was approved for the treatment of RRMS in the European Union in 2013. TFL is a first-line disease-modifying therapy (DMT) with prolonged half-life. Over 21 days, 60.1% of the administered dose is excreted but it may be detectable up to 2 years after discontinuation. The elimination of TFL from the circulation can be expedited using an accelerated elimination procedure (AEP) (2). Based on animal studies, TFL is contraindicated in women who are planning pregnancy due to the occurrence of teratogenicity and embryo lethality in rats and rabbits (3, 4). TFL can be detected in human semen, but the risk of male-mediated embryo fetal toxicity through TFL-treatment is considered low (5). In the United States, the Food and Drug Administration (FDA) advises men to use barrier contraception to prevent the active substance from transferring to their female partner and potentially her fetus. This has not been a requirement in Europe. A recent report (6) presenting post-marketing and clinical study data on 231 female MS patients with TFL-exposed pregnancies (with known outcomes in 129) found no evidence of a teratogenic signal, although three structural abnormalities were reported. Exposure to TFL among the majority of these women had been limited to the first trimester since patients were recommended to undergo an AEP in case of conception.

After discovering the teratogenic link between thalidomide and malformations in more than 10,000 children worldwide, registries were founded to facilitate epidemiological research and surveillance concerning causes of congenital malformation due to genetic, environmental, or medical exposures. Congenital malformations, such as spontaneous abortions, pre-mature birth, physical anomalies, or neurobehavioral outcomes can indicate adverse pregnancy outcomes linked to teratogenic exposure.

The objective of our study was to investigate pregnancy-related outcomes in in TFL-treated female MS patients, in women whose sexual male partners are MS patients treated with TFL registered in the DMSR, and in the new-born of such. Furthermore, we want to investigate any association between TFL exposure during pregnancy and adverse pregnancy-related events.

## Materials and methods

### Registries

All Danish citizens have access to universal health care, which is government-funded through taxation. Most Danish registries have existed for decades, and they are nationwide and population-based, and which provide an excellent foundation for epidemiological research. Persons residing in Denmark have a unique 10 digit ID-number (Civil Registration Number CPR) which can be used to identify and merge data from all Danish registries (7).

#### The danish multiple sclerosis registry (DMSR)

The DMSR was established in 1956 and contains data on all Danes who have been diagnosed by a neurologist as having MS. Data are collected continuously and provide information on a number of baseline and clinical variables (8). Since 1996, a mandatory notification of all MS patients treated with a DMT has been carried out, thus ensuring a high level of data completeness. The registry contains baseline and clinical information on all Danish MS patients with year of onset and diagnosis, disease course, information on DMT, treatment start, treatment stop, previous DMTs and current DMT in addition to first clinical symptom, diagnosis (phenotype), relapses, and Expanded Disability Status Scale (EDSS) (8).

#### The danish medical birth register (DMBR)

The DMBR was established in 1973 and was reconstructed and updated in 1997. The register monitors the health of pregnant women and contains data on all births in Denmark. Several sources feed data into DMBR, such as the Danish National Patient Registry, the Danish Civil Registration System, and data on stillbirths and home deliveries. Data on the mother include height, weight, age, smoking status, and several variables related to the birth, such as pregnancy and birth complications, parity, infection, pain relief during labor, cesarean section, induction of labor etc. In relation to the new-born, it contains a variety of information including sex, gestational age, congenital malformations, Apgar score after 5 min, height, weight, head circumference, and birth status (live birth/stillborn) (9).

#### The danish national patient registry (DNPR)

The DNPR registers every individual episode of a person's contact with hospitals and outpatient clinics in Denmark. It contains multiple information including date of visit, type of contact, examinations, admission type, tests, surgery, treatment, diagnosis, residence, and hospital visited. Diagnoses, surgery, other treatment, anesthesia, and examinations are provided with a code that translates to the standard coding system of the International Coding of Diseases (ICD) (10). The DNPR provides data for several other registries in Denmark including the DMBR and The Register of Legally Induced Abortions. All Danish regions report data to the DNPR, and data are updated at least monthly.

#### The register of legally-induced abortions (RLIA)

The RLIA was established in 1973 when abortion before the end of gestation week 12 became legal in Denmark. The data are mainly from the DNPR, but also includes data from specialized clinicians who carry out legal abortions. Data from the RLIA contain a variety of information including age of the woman, gestational week, prior abortion, type of procedure, legal reason for abortion, complications and admission date (11).

### Data collection

This study was a cohort study comparing outcomes of TFL-treated female MS patients and partners to TFL-treated male patients using prospectively collected nationwide data from the DMSR. The TFL-treated men and women were matched at age of conception 1:4 with controls from the general population. Patients were included from 1st of January 2014 until 31st of December 2016. Included patients should have a confirmed MS diagnosis according to the McDonald criteria, treatment start date with TFL at least 30 consecutive days prior to conception, and conception occurring at the latest 2 years after discontinuation of TFL. The data were merged with the Danish Medical Birth Register, Danish National Patient Registry, and the Register of Legally-Induced Abortions by a unique personal identification number.

In Denmark, pre-term birth is defined as birth before gestation week 37 as described in the literature (12–14). Low birth weight in relation to gestational age was based on the Danish guidelines from the Department of Neonatology, Copenhagen University Hospital (15). Low birthweight for children born after week 37 is defined as a birthweight < 2,500 g, and low birthweight for children born week 33–36 is defined as a birthweight < 2,300 g.

Registration of congenital malformations in Denmark is reported and registered at birth and up to 1 year after birth to allow for delayed identification, as some anomalies are undiagnosed until sometimes after birth e.g., congenital heart disease.

The Apgar score has been used since 1952 and is a quick way to assess the postnatal condition of the new-born. The perspective is to assess for asphyxia, as well as, determine the risk of neurological deficits (16). The assessment is comprised of five components with a maximum of total 10 points, where 7–10 points is considered “reassuring” of normality.

### Controls

The reference cohort was selected randomly from the general population using the Danish Civil Registration System (7), and pregnant women were chosen as controls. We sampled four controls per one TFL-treated man or woman matched by age at conception. Matching of the control population for the female partners of TFL-treated men was done by the age of the man at conception, due to lack of availability of information on the female partners of TFL-treated men.

#### Statistical analysis

Categorical variables were summarized using frequency and percentage. Continuous variables were summarized using mean and standard deviation (SD) or median and interquartile range (IQR) as appropriate. Logistic regression was used to analyse the association between TFL-treated patients and any adverse event (defined as congenital malformation, spontaneous abortion, pre-term birth, or Apgar score < 7), relative to the control group. A Hosmer & Lemeshow test was used to assess the logistic regression for goodness-of-fit. For all statistical comparisons, *p* < 0.05 was considered significant. All analysis was conducted using SAS version 9.4 (SAS Institute Inc., Cary, NC, USA).

## Results

A total of 2,450 TFL-treated patients with MS were identified through the DMSR, of which 31 (18 men and 13 women) met all inclusion criteria. The women had a mean age of 26.6 years (SD 4.9) when diagnosed with MS and 28.5 years (SD 4.7) at conception, while the men had a mean age of 30.7 years (SD 6.7) at diagnosis and 34.1 years (SD 3.9) at conception (Tables 1, 2). In the female study group, 7 of 13 (53.8%) were being treated with TFL at conception, with an average treatment period of 154 days (SD 111.3). The remaining six conceived on average 316 days (SD 234.8) after discontinuation. Two TFL treated women decided to continue their pregnancy to term, one conceived 35 days after discontinuation of TFL (352 total days on TFL), and the other 95 days after discontinuation of TFL (179 total days on TFL). Only one man fathered a child after discontinuing TFL (343 days later), while the remaining 17 men had been treated with TFL an average of 198 days (SD 148.2 days) at child conception (Table 2).

**Table 1:** Baseline characteristics of patients in the study group *n* = 31 (MS^*^ patients exposed to TFL^**^ >30 consecutive days when becoming/partner becoming pregnant and up to 2 years after discontinuation of TFL treatment), and the reference group (*n* = 124).

	**Study group** ***n*** = **31**		**Reference group** ***n*** = **124**	
	**Male**	**Female**	**Male**	**Female**
*N* (%)	18 (59%)	13 (41%)	72 (59%)	52 (41%)
**YEAR OF BIRTH**, ***n***				
1965–1984	16	3	64	23
1985–2002	2	10	8	29
**AGE AT ONSET IN YEARS**, ***n***				
0–24	4	6	N/A^***^	N/A
25–34	11	6	N/A	N/A
35–44	3	1	N/A	N/A
Mean (min;max)sd^***^	29.16 (12;38)6.76	25.23 (16;36)5.18	N/A	N/A
**AGE AT DIAGNOSIS IN YEARS**, ***n***				
0–24	2	5	N/A	N/A
25–34	9	7	N/A	N/A
35–44	7	1	N/A	N/A
Mean (min;max)sd^****^	30.72 (13;39)6.79	26.61 (19;37)4.91	N/A	N/A

* *Multiple sclerosis*.

** *Teriflunomide*.

*** *Not applicable*.

**** *Standard deviation*.

**Table 2:** Data regarding conception within the study group *n* = 31 (MS^*^ patients exposed to TFL^**^ >30 consecutive days when becoming/partner becoming pregnant and up to 2 years after discontinuation of TFL treatment), and the reference group (*n* = 124).

	**Study group** ***n*** = **31**		**Reference group** ***n*** = **124**	
	**Male (*n* = 18)**	**Female (*n* = 13)**	**Male (*n* = 72)**	**Female (*n* = 52)**
**AGE AT CONCEPTION**				
19–24	0	3	0	12
25–30	3	6	13	24
31–35	10	3	40	12
36–40	3	1	12	4
>40	2	0	8	0
Mean (min;max)sd^***^	34.1 (27;42)3.9	28.5 (19;38)4.8	34.1 (27;42)3.9	28.5 (19;38)4.6
**DURATION ON TFL AT CONCEPTION IN DAYS**				
31–90	5	2	N/A^****^	N/A
91–180	4	3	N/A	N/A
181–365	5	1	N/A	N/A
>365	3	1	N/A	N/A
Mean (min;max)sd	198 (34;463)148.1	154 (47;380)111.3	N/A	N/A
**CONCEPTION AFTER TREATMENT STOP IN DAYS**				
31–90	0	1	N/A	N/A
91–180	0	1	N/A	N/A
181–365	1	2	N/A	N/A
366–730	0	2	N/A	N/A
Mean (min;max)sd^***^	343	316.5 (35;688)234.8	N/A	N/A

* *Mulitple sclerosis*.

** *Teriflunomide*.

*** *Standard deviation*.

**** *Not applicable*.

A total of 31 pregnancies were included in the analysis, matched to 124 controls. Of the 31 pregnancies comprising the study group, 13 were women treated with TFL whilst the remaining 18 were female partners of TFL-treated men. All 18 female partners of TFL-treated men completed their pregnancies resulting in live births. Of these, 17 had a normal gestation time (>37 weeks) and one gave birth in week 36. All new-borns recorded a normal birth weight relative to gestation week, and no spontaneous or elective abortions were reported. 88.89% (16 out of 18) of the female partners to a TFL-treated man had vaginal delivery, of which two were induced, and the remaining two had cesarean section (Table 3). All 18 new-borns had Apgar score ≥7, and one new-born had a congenital malformation (plagiocephaly) (Table 4).

**Table 3:** Characteristics of delivery outcomes; namely still or live birth, abortions and delivery mode in the study group *n* = 31 (MS^*^ patients exposed to TFL^**^ >30 consecutive days when becoming/partner becoming pregnant and up to 2 years after discontinuation of TFL treatment), and the reference group (*n* = 124).

	**Study group (*****n*** = **31)**		**Reference group (*****n*** = **124)**	
	**Male (*n* = 18)**	**Female (*n* = 13)**	**Male (*n* = 72)**	**Female (*n* = 52)**
**BIRTHS**				
Still births	0	0	0	0
Live births	18	2	72	37
**ABORTIONS**				
Elective	0	11	0	12
Spontaneous	0	0	0	3
**DELIVERY**				
Normal birth singleton	14	1	42	24
Normal birth multiples	0	0	1	0
Induced	2	1	19 (1 missing)	5
**CESAREAN**				
Acute	2	0	5	6
Elective	0	0	4	2

* *Multiple sclerosis*.

** *Teriflunomide*.

**Table 4:** Characteristics of birth-related outcome; namely gestation time, birth weight in relation to gestation time, Apgar score, and congenital malformations in the study group *n* = 31 (MS^*^ patients exposed to TFL^**^ >30 consecutive days when becoming/partner becoming pregnant and up to 2 years after discontinuation of TFL treatment), and the reference group (*n* = 124).

	**Study group (*****n*** = **31)**		**Reference group (*****n*** = **124)**	
	**Male (*n* = 18)**	**Female (*n* = 13)**	**Male (*n* = 72)**	**Female (*n* = 52)**
**GESTATION TIME**				
Normal >37 weeks	17	2	67	34
Pre-term birth < 37 weeks	1	0	5	3
**BIRTH WEIGHT IN RELATION TO GESTATIONAL WEEK**				
Normal	18	2	68	37
Low	0	0	3	0
**APGAR SCORE**				
Low < 7	0	0	0	0
Normal ≥7	18	2	71 (1 missing)	37
**CONGENITAL MALFORMATIONS**				
Plagiocephaly	1	0	1	0
Cardiac septum	0	0	1	0
Respiratory system	0	0	0	1

* *Multiple sclerosis*.

** *Teriflunomide*.

Among the 13 pregnant women treated with TFL, 11 (85%) chose an elective abortion. There were no reports of spontaneous abortions. Two women chose to continue the pregnancy to term, both of which resulted in live births. Both pregnancies were carried to term with normal gestation time >37 weeks, normal birth weight, one vaginal delivery, and one induced. Both new-borns had an Apgar score ≥7 with no reports of congenital malformations (Tables 3, 4).

The study group had two adverse events, namely one congenital malformation and one pre-term birth (week 36). The comparison group presented 14 adverse events, namely three spontaneous abortions, eight pre-term deliveries, and three congenital malformation (Table 4). Stillbirth has not been included as an adverse event, as no such event occurred in either of the groups. The study group was associated with a 22% reduction in the odds of any adverse event relative to controls, although this association was not significant (OR 0.78; 95% CI 0.16–3.72, *p* = 0.753).

## Discussion

In this nationwide population-based study, we did not find any deviations in either women or new-borns among female patients treated with TFL or in women of male partners treated with TFL in terms of spontaneous abortions, pre-term delivery (< week 37), low Apgar score (< 7), or congenital malformations when compared to the general population. Elective abortions among women treated with TFL were unsurprisingly higher, although the two women who continued the pregnancy had healthy live births. None of the female partners of the TFL-treated men chose an elective abortion.

Overall, studies investigating the perinatal characteristics and obstetric complications in mothers with MS compared to the background population have not found any differences in either neonatal or obstetric complications (17, 18), although one study found a slightly higher rate of induced labor and cesarean delivery among women with MS than in the reference group (19).

In general, female MS patients who intend to get pregnant are advised to discontinue their DMT based on the potential risk of adverse events in relation to DMT exposure during pregnancy. However, this might result in a worsening of the disease (20).

Teriflunomide is contraindicated in women who are pregnant and women of childbearing potential who are not using effective contraception during treatment based on preclinical findings of embryotoxicty and teratogenicity in rats and rabbits when treated with clinically relevant doses of TFL. Later research showed a difference in the affinity of dihydroorotate dehydrogenase for TFL between rats and humans. Thus, TFL is a more potent inhibitor of the rat enzyme than the human enzyme, which may reflect the observed embryotoxicity and teratogenicity in rats (21–23). In Europe, it is not advised against fathering children during TFL treatment. Also, although women are required to use reliable contraception when treated with TFL, pregnancies in women treated with TFL have been reported.

In Denmark, the estimated incidence of spontaneous abortions in the general population is 13.5% (24), and the incidence of pre-mature new-born (< 37 weeks) has been stable at 6% during the last 5 years based on numbers from the Danish National Birth Register (25). In this study, there were no cases of registered spontaneous abortions among the TFL study group, and one pre-term birth in the TFL-exposed group equivalent to an incidence rate of 5.5%; corresponding to that of the general population.

Previous research investigating pregnancy-related outcomes of mother and new-born in relation to TFL exposure (6, 26) reported a higher incidence rate for spontaneous abortions in the TFL-treated patients, respectively, 18.6 and 21%, respectively, than that reported for the general population in Denmark. Kieseier et al. reported a mean of gestation week 39 (range 36–44 weeks) among women treated with TFL equivalent to what is reported in this study (both >week 37).

Plagiocephaly, the congenital malformation reported in the TFL-exposed group in this study, is also known as “flat head syndrome.” It is a condition characterized by an asymmetrical distortion of the infant's skull. It is important to differentiate between craniosynostosis and positional skull deformities, also known as synostotic and non-synostotic plagiocephaly (27). In case of skull deformity at birth, a pediatrician should differentiate whether the cause is synostotic, as synostotic plagiocephaly worsens over time, causes severe complications, and often requires surgery. Deformational plagiocephaly (DP), or non-synostotic plagiocephaly, is normally benign and the most common cause of plagiocephaly with prevalence of 5–48% in healthy new-borns (27, 28). It can be caused when the child passes through the birth canal, or due to gravitational forces, such as the dramatic increase in DP that was seen in the 1990's after implementing the recommendation for infants to sleep in a supine position to decrease the risk of sudden infant death syndrome.

Kieser et al. found no structural or functional abnormalities at birth among new-borns of either TFL treated women or in women whose male partners were treated with TFL. It is not mentioned if abnormalities appearing after birth were considered. Vukusic et al. reported one fetal death ≥20 weeks of gestation, and three structural abnormalities, but without comparison of the results to any reference group or general population. Both previous studies conclude no evidence of teratogenic signals in TFL-treated women or in females whose male partners were treated with TFL.

A strength of this study is the use of nationwide population-based data, although the small sample size is a limitation in terms of generalizations or absolute recommendations. The incidence of spontaneous abortion, pre-term delivery, or congenital malformation in female partners of TFL-treated men and TFL-treated women is no different than that of the general Danish population. A possible limitation of this study is the unknown age of the female partners of the men treated with TFL.

## Conclusion

The post-marketing analyses of this report, based on nationwide register-based data from the availability of TFL in Denmark until 1st of January 2017 do not indicate teratogenicity due to TFL in pregnancy or pregnancy-related outcomes. This is in line with the known outcomes from both clinical trials and post-marketing studies.

Further analyses of available pregnancy data with TFL are warranted to better quantify the risk associated with TFL exposure in pregnancy before concluding against teratogenicity.

## Data availability statement

The raw datasets used for this study have been obtained from a third party, the Danish Data Protection agency, based upon written approval of the study protocol. Request to access the datasets will require an individual inquiry to the Danish Data Protection agency for approval based on the Danish legislation.

## Author contributions

JA study concept and design, data acquisition, statistical analyses, interpretation of the results, drafting, and revision of the manuscript. JM study concept and critical revision of the manuscript. TS revision of the manuscript and statistical analyses. MM study concept and design, data acquisition, interpretation of results, revision of the manuscript.

### Conflict of interest statement

JA has received travel grants and support for congress participation from Merck. JM has served on scientific advisory board for Biogen, has received speaker honoraria from Biogen, Sanofi Genzyme, and Teva, and has received support for congress participation from has Biogen, Merck, Roche, Sanofi Genzyme, and Teva. TS received compensation for serving on scientific advisory boards, honoraria for consultancy and funding for travel from Biogen; speaker honoraria from Novartis. MM has served on scientific advisory board for Biogen, Sanofi, Teva, Roche, Novartis, Merck, has received honoraria for lecturing from Biogen, Merck, Novartis, Sanofi, has received support for congress participation from Biogen, Genzyme, Teva, Roche.

## References

[1] Lublin FD. New multiple sclerosis phenotypic classification. Eur Neurol. (2014) 72:1–5. 10.1159/000367614 25278115

[2] Freedman MS. Teriflunomide in relapsing multiple sclerosis: therapeutic utility. Ther Adv Chronic Dis. (2013) 4:192–205. 10.1177/2040622313492810 23997924 PMC3752181

[3] Cada DJ Demaris K Levien TL Baker DE. Teriflunomide. Hosp Pharm. (2013) 48:231–40. 10.1310/hpj4803-231 24421467 PMC3839508

[4] FDA Aubagio [Package Insert]. Cambridge, MA: Genzyme Corporation (2012). Available online at: https://www.accessdata.fda.gov/drugsatfda_docs/label/2012/202992s000lbl.pdf

[5] Kieseier BC Benamor M. Pregnancy outcomes following maternal and paternal exposure to teriflunomide during treatment for relapsing–remitting multiple sclerosis. Neurol Ther. (2014) 3:133–8. 10.1007/s40120-014-0020-y 26000229 PMC4386431

[6] Vukusic S Coyle PK Jurgensen S Truffinet P Benamor M Poole E Pregnancy outcomes in patients with MS treated with teriflunomide: clinical study and postmarketing data (P.4.361). Neurol. Adv. Commun. (2018) 90:202563. Available online at: http://onlinelibrary.ectrims-congress.eu/ectrims/2017/ACTRIMS-ECTRIMS2017/202563/sandra.vukusic.pregnancy.outcomes.in.patients.with.ms.treated.with.html 10.1177/1352458519843055 30968734

[7] Schmidt M Pedersen L Sørensen HT. The Danish civil registration system as a tool in epidemiology. Eur J Epidemiol. (2014) 29:541–9. 10.1007/s10654-014-9930-3 24965263

[8] Magyari M Koch-Henriksen N Sørensen PS. The danish multiple sclerosis treatment register. Clin Epidemiol. (2016) 8:549–52. 10.2147/CLEP.S99500 27822098 PMC5094645

[9] Bliddal M Broe A Pottegård A Olsen J Langhoff-Roos J. The Danish medical birth register. Eur J Epidemiol. (2018) 33:27–36. 10.1007/s10654-018-0356-1 29349587

[10] Schmidt M Schmidt SAJ Sandegaard JL Ehrenstein V Pedersen L Sørensen HT. The Danish national patient registry: a review of content, data quality, and research potential. Clin Epidemiol. (2015) 7:449–90. 10.2147/CLEP.S91125 26604824 PMC4655913

[11] Tølbøll Blenstrup L Knudsen LB Danish registers on aspects of reproduction. Scand J Public Health (2011) 39:79–82. 10.1177/1403494811399957 21775359

[12] Tsimis ME Abu Al-Hamayel N Germaine H Burd I. Prematurity: present and future. Minerva Ginecol. (2015) 67:35–46. 25300768 PMC4323881

[13] Quinn JA Munoz FM Gonik B Frau L Cutland C Mallett-Moore T. Preterm birth: case definition & guidelines for data collection, analysis, and presentation of immunisation safety data. Vaccine (2016) 34:6047–56. 10.1016/j.vaccine.2016.03.045 27743648 PMC5139808

[14] Mazaki-Tovi S Romero R Kusanovic JP Erez O Pineles BL Gotsch F. Recurrent preterm birth. Semin Perinatol. (2007) 31:142–58. 10.1053/j.semperi.2007.04.001 17531896 PMC3500643

[15] Videnscenter R Definitioner for Tidligt Født – Riget. Available online at: https://www.rigshospitalet.dk/afdelinger-og-klinikker/julianemarie/videnscenter-for-tidligt-foedte-boern/om-tidligt-foedte/Sider/Definitioner.aspx

[16] American Academy of Pediatrics Committee on Fetus and Newborn Use and abuse of the apgar score. Pediatrics. (1996) 98:141–2. 3786042

[17] Goldacre A Pakpoor J Goldacre M. Perinatal characteristics and obstetric complications in mothers with multiple sclerosis: record-linkage study. Mult Scler Relat Disord. (2017) 12:4–8. 10.1016/j.msard.2016.11.011 28283105

[18] Yalcin SE Yalcin Y Yavuz A Akkurt MO Sezik M. Maternal and perinatal outcomes in pregnancies with multiple sclerosis: a case-control study. J Perinat Med. (2017) 45:455–60. 10.1515/jpm-2016-0060 27124670

[19] Fong A Chau CT Quant C Duffy J Pan D Ogunyemi DA. Multiple sclerosis in pregnancy: prevalence, sociodemographic features, and obstetrical outcomes. J Matern Neonatal Med. (2018) 31:382–7. 10.1080/14767058.2017.1286314 28139946

[20] Fragoso YD Boggild M MacIas-Islas MA Carra A Schaerer KD Aguayo A. The effects of long-term exposure to disease-modifying drugs during pregnancy in multiple sclerosis. Clin Neurol Neurosurg. (2013) 115:154–9. 10.1016/j.clineuro.2012.04.024 22633835

[21] Davenport L Edling A Finn P Truffinet P Teriflunomide Mechanism of Action: Linking Species' Sensitivities to Pregnancy Outcomes. Vol. 23 Copenhagen: 29th Congress of the European Commitee for Treatment Research in Multiple Sclerosis, 2013 (2015). Available online at: http://ovidsp.ovid.com/ovidweb.cgi?T=JS&PAGE=reference&D=emed17&NEWS=N&AN=72058744

[22] Cassina M Johnson DL Robinson LK Xu R Jimenez JL Mirrasoul N Pregnancy outcome in women exposed to leflunomide before or during pregnancy. Arthritis Rheum. (2012) 64:2085–94. 10.1002/art.34419 22307734

[23] Alwan S Chambers CD Armenti VT Sadovnick AD. The need for a disease-specific prospective pregnancy registry for multiple sclerosis (MS). Mult Scler Relat Disord. (2015) 4:6–17. 10.1016/j.msard.2014.10.001 25787048

[24] Andersen A-MN Maternal age and fetal loss: population based register linkage study. BMJ. (2000) 320:1708–12. 10.1136/bmj.320.7251.1708 10864550 PMC27416

[25] The Danish National Birth Register. Available online at: http://www.esundhed.dk/sundhedsregistre/MFR/Sider/MFR06A.aspx

[26] Kieseier B Truffinet P Jung Henson LBM Pregnancy outcomes for female patients and partners of male patients in the teriflunomide clinical development program. Mult Scler. (2014) 20(1 Suppl. 1):438 10.1016/j.msard.2014.09.19224005026

[27] Ghizoni E Denadai R Raposo-Amaral CA Joaquim AF Tedeschi H Raposo-Amaral CE. Diagnosis of infant synostotic and nonsynostotic cranial deformities: a review for pediatricians. Rev Paul Pediatr. (2016) 34:495–502. 10.1016/j.rppede.2016.02.005 27256993 PMC5176072

[28] Losee JE Mason AC. Deformational plagiocephaly: diagnosis, prevention, and treatment. Clin Plast Surg. (2005) 32:53–64. 10.1016/j.cps.2004.08.003 15636765

